# Impact of coronavirus disease 2019 on consumers’ perceptions of genetically modified food

**DOI:** 10.1080/21645698.2023.2204051

**Published:** 2023-04-23

**Authors:** Qian Ding, Fangbin Qiao, Jikun Huang

**Affiliations:** a China Economics and Management Academy, Central University of Finance and Economics, Haidian, Beijing, China; b China Center for Agricultural Policy, Peking University, Haidian, Beijing, China

**Keywords:** Consumer perception, COVID-19, genetically modified food, purchase behavior, purchase intention

## Abstract

The coronavirus disease 2019 (COVID-19) pandemic has generated significant economic loss and an unprecedented challenge to people’s livelihoods. Using household data collected in November 2020, this study shows that the COVID-19 outbreak has significantly affected consumers’ perceptions and consumption of genetically modified (GM) food in China. Their perceptions and purchase intentions have turned more negative, and their actual purchase of GM food has decreased after the COVID-19 outbreak. The study’s results also indicate that consumers with more knowledge of genetic modification technology are less likely to change their perceptions of GM food.

## 1. Introduction

The outbreak of the coronavirus disease 2019 (COVID-19) has significantly impacted household income and consumption patterns.^1–3^ For example, previous studies have shown that consumers’ total spending on food has decreased,^4^ while online shopping has substantially increased during the COVID-19 pandemic.^5^ Other studies have shown that the importance of food safety attributes has increased dramatically after the COVID-19 outbreak,^6^ and consumers’ purchase intentions for organic food have increased.^7^

However, few studies focus on genetically modified (GM) food, even though it is at the center of a heated debate.^8,9^ Genetic modification technology is a highly controversial topic for global food consumers.^10^ Although no scientific research has unambiguously proven that GM food may cause adverse health issues,^11^ concerns about the safety of GM products continue to emerge.^12^ The negative perception of GM food has become predominant in China since the first decade of the 2000s,^10^ although more than half of Chinese consumers had positive perceptions of GM food two decades ago.^13,14^

This study assesses the impact of COVID-19 on consumers’ perceptions of GM food safety. First, we examine whether the COVID-19 outbreak has significantly changed consumers’ perceptions and consumption of GM food. Second, we investigate various factors affecting consumers’ change in attitudes toward GM food, exploring whether consumers’ knowledge of GM technology is associated with a significant attitude change after the COVID-19 outbreak.

## 2. Methodology and Data

### 2.1 Field Survey

To examine consumers’ perceptions and consumption of GM food before and after the COVID-19 outbreak, we conducted a field survey in November 2020. The survey covers nine cities in six provinces across China: Harbin in Heilongjiang province (Northeast China), Lanzhou in Gansu province (Northwest China), Beijing (North China), Jinhua and Ningbo in Zhejiang province, Nanjing and Yancheng in Jiangsu province (East China), and Guangzhou and Zhongshan in Guangdong province (South China). We select these nine cities from the Urban Household Income and Expenditure Surveys conducted by the National Bureau of Statistics of China,^9^ collecting data from 2030 consumers (last row of Table 1).

**Table 1.** Basic statistics of major variables used in this study.

Variable names	Mean	Standard deviation
**Perception of GM food**		
Strongly approve	0.03	0.18
Approve	0.18	0.38
Neutral	0.38	0.49
Oppose	0.23	0.42
Strongly oppose	0.17	0.38
**Purchase intention**		
Strongly approve	0.03	0.17
Approve	0.17	0.38
Neutral	0.33	0.47
Oppose	0.30	0.46
Strongly oppose	0.16	0.37
**Actual purchase (yes = 1)**	0.13	0.33
**Consumers’ characteristics**		
Male (yes = 1)	0.44	0.50
Age	38.70	14.78
High school and above (yes = 1)	0.77	0.42
Government or state-owned firm employee	0.10	0.30
Private company employee	0.53	0.50
Student	0.12	0.32
Other jobs	0.26	0.44
Agriculture-related job	0.06	0.24
Number of years the consumer has been aware of GM food	7.77	4.17
Grocery shopping	0.49	0.50
**Household characteristics**		
Family size	3.49	1.35
Low income^a^	0.14	0.35
Middle income^a^	0.43	0.49
High income^a^	0.28	0.45
Family member with a food allergy (yes = 1)	0.29	0.46
Large city (yes = 1)	0.32	0.47
Medium-sized city (yes = 1)	0.47	0.50
Small city (yes = 1)	0.21	0.41

Note: ^a^ “Low-income” families are those whose total family income is less than 50,000 yuan per year; high-income families are those whose total family income is more than 100,000 yuan per year, and middle-income families are those whose total family income is between 50,000 and 100,000 yuan per year. GM: genetically modified.

We adopt several measures to improve the field survey’s data quality. First, this study collects data through in-person interviews, even though online surveys have mostly replaced traditional surveys after the COVID-19 outbreak. Doing so may avoid potential estimation bias due to survey fraud and sample response bias. Second, to avoid potential selection bias, depending on the individuals interviewed in each household, we ask interviewers to interview the first adult (aged 16–70) they meet upon arriving at the interviewee’s apartment. Finally, to reduce potential estimation bias due to measurement errors, we employ trained graduate students and professional researchers, and we adapt most questionnaire items from previous studies (e.g., Huang and Peng).^9^

The questionnaire comprises several sections. The first section records essential household characteristics, such as family size and income, and explores whether family members have ever experienced food allergies. The second section records respondents’ demographic information (e.g., gender, age, education, job category, and whether they are in charge of grocery shopping). Table 1 summarizes the household and individual characteristics.

Finally, the questionnaire records consumers’ perceptions and consumption of GM food.^1^^1^Both indirectly processed GM agricultural products and products made with agricultural GMOs as raw materials, such as soybean oil, should be labeled in accordance with the Agricultural Genetically Modified Organisms Identification Management Measures issued in 2002.^15^. To this end, we ask respondents to assess their perceptions of GM food, providing them with five choices: (1) I strongly approve, (2) I approve, (3) I am neutral, (4) I oppose, and (5) I strongly oppose.^2^^2^For simplicity, respondents answering “no idea” or “I do not know” are also classified into the neutral group. By considering it as a new group and replacing the ordered logit model with the multinomial logit model, we rerun all the models and obtain similar results. Similarly, we classify respondents’ purchase intentions into five categories. Finally, we ask respondents whether they purchase any of three GM foods: papaya, soybean oil, and tofu. Respondents answered these three questions in 2019 (before the COVID-19 outbreak) and 2020 (after the COVID-19 epidemic).

### 2.2 Consumers’ Perceptions and Consumption of GM Food Before and After the COVID-19 Outbreak

**Figure 1.**
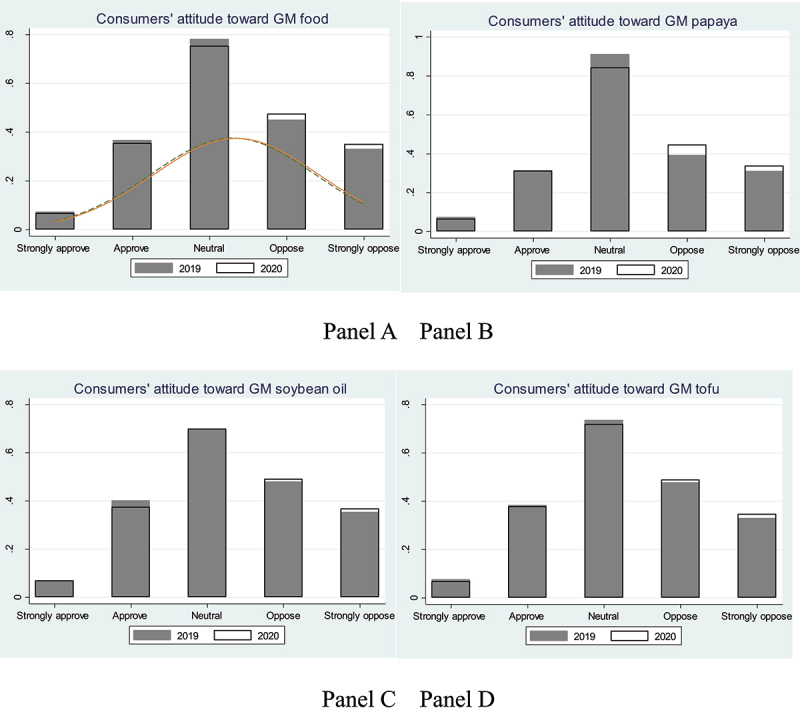
Consumers’ perceptions of genetically modified food before and after the COVID-19 outbreak.

Note: GM: genetically modified.

We compare consumers’ perceptions and consumption of GM food between 2019 and 2020. As shown in Panel A of Fig. 1, compared to 2019, the share of consumers with a strongly positive, positive, and neutral perception of GM food has significantly reduced in 2020, after the COVID-19 outbreak. By contrast, the share of consumers with negative or strongly negative perceptions has increased in 2020. Fig 1 shows that consumers’ perceptions of GM food have turned negative after the COVID-19 outbreak. Approximately 11% of consumers have changed their perceptions after COVID-19. Among them, 63% have become more risk-averse, opposing GM food. This finding is robust to the choice of three different GM foods (i.e., papaya, soybean oil, and tofu), as shown in Panels B – D of Fig. 1.

**Figure 2.**
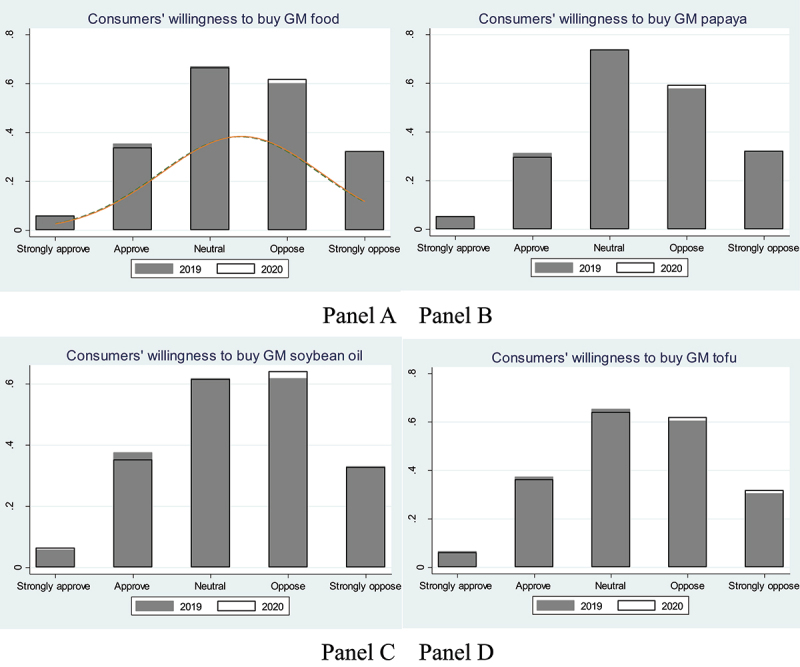
Consumers’ intentions to purchase genetically modified food before and after the outbreak of COVID-19.

Note: GM: genetically modified.

In line with the change in perception, consumers’ purchase intentions have also become more negative (i.e., opposing GM food) after the COVID-19 outbreak. As shown in Panel A of Fig. 2, the share of consumers with positive purchase intentions toward GM food (i.e., approve and strongly approve) has decreased, while the share of consumers with negative purchase intentions (i.e., oppose and strongly oppose) has increased after the COVID-19 outbreak. Less than 5% of consumers’ purchase intentions have increased, while more than 6% have decreased during the COVID-19 pandemic. This finding is robust to the choice of three different GM foods (Panels B – D of Fig. 2).

**Figure 3.**
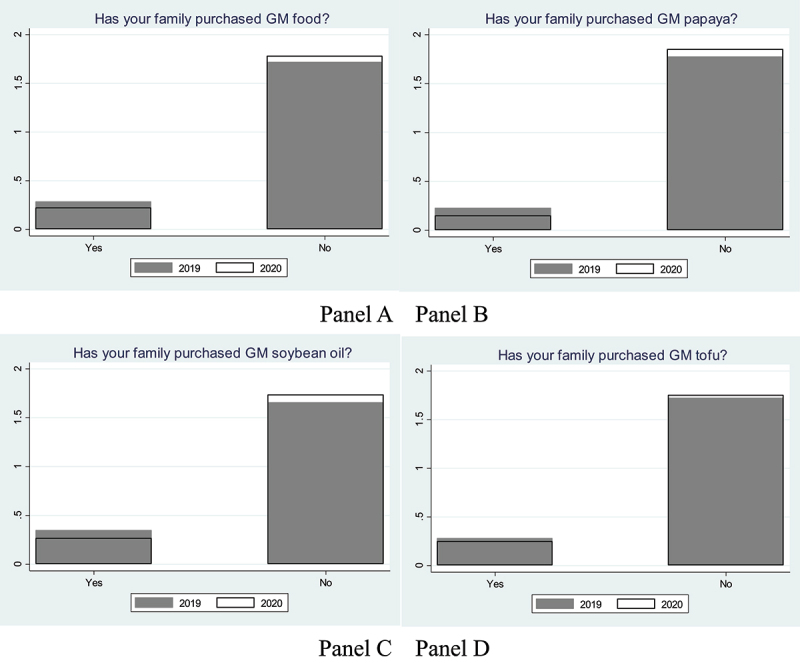
Consumer’s actual purchase of GM food before and after the COVID-19 outbreak.

Note: GM: genetically modified.

Finally, Fig. 3 shows that fewer consumers have purchased GM food in 2020, after the COVID-19 outbreak (Panels A – D). Our data show that 14% of consumers have purchased GM food in 2019, while only 11% have purchased it in 2020. In other words, 22% of consumers who have purchased GM food in 2019 have changed their consumption behaviors after the COVID-19 outbreak.

### 2.3 Estimation Strategy

#### 2.3.1 Impact of COVID-19

Fig 1–3 may be misleading because other factors affect these results. To disentangle the impact of the COVID-19 outbreak, we estimate a multivariate regression as follows:

(1) $$Perception_{ij}=\alpha_{0}+\alpha_{1}COVID-19_{i}+\alpha_{2}Individual_{i}+\alpha_{3}Household_{i}+\alpha_{4}Other_{ij}+e_{ij}$$

(1) Three variables measure consumers’ perceptions and consumption of GM food (*Perception*): 1) perception, 2) purchase intention, and 3) actual purchase.^3^^3^We did not consider the availability of GM products after the outbreak of COVID-19. However, we believe that this limitation would not result in significant estimation bias since none of the sampled cities had been placed under full or partial COVID-19 lockdowns before we conducted the field survey. Subscript *i* indicates the *i*th individual, *j* is the *j*th product (papaya, soybean oil, and tofu), and *t* = 2019 and 2020; *e* is the error term.

The first independent variable is *COVID-19*, a dummy variable, which equals one in 2020 (after the COVID-19 outbreak), and zero in 2019 (before COVID-19). The estimated coefficient on this variable indicates whether consumers’ perceptions and consumption behaviors have changed after the COVID-19 outbreak; a significant coefficient indicates a change.

Equation (1) also incorporates two vector variables, *Individual* and *Household*. The *Individual* vector includes the number of years a consumer has been aware of GM food, the gender of the respondent (male equals one, and female equals zero), a high school and above education dummy, three occupation dummies (i.e., working in a government organization or state-owned enterprise, working in a private enterprise, student and others), and whether the respondent is in charge of grocery shopping for the family.

Similarly, the *Household* vector includes household size and family income. To consider the impact of food safety, we also include a dummy variable indicating whether any family members have ever experienced food allergies. This variable equals one if any family members have experienced food allergies, and zero otherwise.

Finally, we add the *Other* vector variable to assess the impact of other factors that affect consumers’ perceptions and consumption of GM food. For example, consumers’ perceptions may be affected by city size. To capture the impact of city size, we include two dummy variables, one for medium-sized cities (taking a value of one for medium-sized cities, and zero otherwise) and one for large cities (assuming a value of one for large cities, and zero otherwise).

When we employ consumers’ perceptions and purchase intentions toward GM food as the dependent variables, we estimate Equation (1) using an ordered logit (ologit) regression model. Similarly, when we use whether consumers purchase GM food as the dependent variable, we estimate Equation (1) using a logit regression model. The estimated parameters of the ologit and logit models indicate the nature of the impact but do not measure the quantitative marginal impact of the independent variables. Hence, we then estimate the marginal effects after estimating the ordered logit and logit models.

Even though we control the individual and household characteristics in Equation (1), some unobserved variables (such as consumers’ preferences) may still be omitted, generating biased results.^4^^4^Even though the results from Equation (1) may be biased, we estimate Equation (1) and discuss the estimation results (Tables 2 and 3) for two reasons. First, most previous studies ignore the endogeneity caused by omitted variables. Following this praxis, we compare our estimation results to those obtained by previous studies. Second, as shown in the next section, we obtain similar results by excluding the impact of time-invariant variables from the estimation (i.e., Equation 2).

**Table 2.** Impact of COVID-19 on consumers’ perceptions and consumption behavior.

	Dependent variable		
	perception	purchase intention	actual purchase
COVID-19 (Yes = 1)	0.0832***	0.0407***	−0.3135***
	(5.02)	(2.63)	(−4.81)
Knowledge of GM	−0.4492***	−0.4472***	0.2372***
	(−19.60)	(−18.27)	(5.46)
Number of years the consumer has been	0.0659***	0.0644***	0.0015
aware of GM food	(8.69)	(5.68)	(0.16)
Male (1=male)	−0.1504***	−0.1424**	0.3386***
	(−2.88)	(−2.41)	(3.67)
Government or state-owned firms	−0.0894	−0.0974	0.2332
(Yes = 1)	(−0.59)	(−0.61)	(1.17)
Private firms (Yes = 1)	−0.3464	−0.3140	0.0178
	(−1.59)	(−1.44)	(0.09)
Student (Yes = 1)	0.2079	0.2092	−0.0063
	(1.09)	(1.08)	(−0.03)
Agriculture-related work (Yes = 1)	−0.4778***	−0.4179**	0.5094***
	(−3.48)	(−2.42)	(4.79)
Grocery shopping dummy	0.1262*	0.1589*	0.1388*
	(1.69)	(1.65)	(1.82)
Family size	0.0189	0.0319	0.0582
	(0.49)	(0.76)	(1.15)
Family member with a food allergy	0.1978	0.1484	0.1246
	(1.53)	(1.46)	(0.78)
Middle-income (50 ~ 100k) dummy	−0.1555	−0.1294	0.1331
	(−1.43)	(−1.25)	(1.62)
High-income (>100k) dummy	−0.0878	−0.0856	0.1429
	(−0.74)	(−0.73)	(1.38)
Soybean oil dummy	0.0710***	0.0032	0.5976***
	(2.87)	(0.10)	(3.89)
tofu dummy	0.0226	−0.0746**	0.4019*
	(0.72)	(−2.16)	(1.89)
Nanjing city dummy	0.4650***	0.3282***	0.4008***
	(14.34)	(10.27)	(8.75)
Yancheng city dummy	0.4281***	0.3701***	0.1817***
	(10.97)	(8.78)	(3.64)
Ningbo city dummy	−0.2299***	−0.3397***	0.7983***
	(−3.80)	(−5.69)	(12.47)
Jinhua city dummy	−0.0619	−0.2123***	−0.2642***
	(−1.12)	(−4.19)	(−3.52)
Guangzhou city dummy	−0.4408***	−0.5216***	0.4057***
	(−8.25)	(−11.35)	(7.59)
Zhongshan city dummy	−0.4359***	−0.5031***	−1.0704***
	(−7.73)	(−9.85)	(−16.55)
Lanzhou city dummy	−0.3376***	−0.3202***	−0.4518***
	(−5.55)	(−4.99)	(−6.68)
Beijing city dummy	−0.1863***	−0.2491***	−0.3858***
	(−4.30)	(−6.29)	(−9.31)
Constant			−3.3325***
			(−15.24)
Observations	12,180	12,180	12,180

Note: City cluster robust z-statistics in parentheses. ***p < .01, **p < .05, *p < .1. GM: genetically modified.

**Table 3.** Marginal effect of COVID-19 on consumers’ perceptions and consumption of genetically modified food.

	perception	purchase intention	actual purchase
Strongly approve	−0.0029***	−0.0012***	
	(−4.32)	(−2.62)	
Approve	−0.0092***	−0.0045***	
	(−4.47)	(−2.60)	
Neutral	−0.0043***	−0.0025**	
	(−5.55)	(−2.47)	
Oppose	0.0058***	0.0032**	
	−5.88	−2.48	
Strongly oppose	0.0106***	0.0050***	
	−4.17	−2.61	
Yes			−0.0336***
			(−4.76)
Observations	12,180	12,180	12,180

Note: z-statistics in parentheses. ***p < .01, **p < .05, *p < .1.),” To solve the potential estimation bias due to endogeneity, we estimate a fixed-effect model as follows:

(2) $$\begin{array}{l} Perception_{ij}=\vartheta_{0}+\vartheta_{1}COVID-19_{i}\\ \;\;\;\;\;\;\;\;\;\;\;\;\;\;\;\;\;\;\;\;\;\;\;\;\;\;\;\;\;\;+\sum_{j=1}^{j=3}\left(\vartheta_{2j}Product_{i}+\vartheta_{3j}Product_{i}{}^{*}COVID-19_{i}\right)\\ \;\;\;\;\;\;\;\;\;\;\;\;\;\;\;\;\;\;\;\;\;\;\;\;\;\;\;\;\;\;+\sum_{i=1}^{i=N-1}ID_{i}+\tau ijt \end{array}$$

In Equation (2), we control the impact of unobserved and time-consistent factors by adding individual dummies (i.e., *ID*_*i*_).^5^^5^Due to data availability, the impact of other variables that vary between 2019 and 2020, such as income, is omitted. Previous studies show that the COVID-19 outbreak has reduced income.^16,17^ In addition, consumers’ perceptions and consumption of GM food become negative as income increases.^9^ Hence, the omitted variables may lead to an underestimation of the impact of COVID-19. Finally, as we measure consumers’ perceptions and purchase intentions using a five-point Likert scale, we estimate Equation (2) using a fixed-effect ologit model. Similarly, we estimate a fixed-effect logit model to identify factors affecting consumers’ consumption behaviors (i.e., actual purchase).

#### 2.3.2 Determinants of consumers’ Attitude Shift

We also explore various factors affecting these changes. By identifying these factors, we provide suggestions to mitigate the impact of COVID-19. The econometric model used to determine the factors influencing consumers’ changes is very similar to Equation (1):

(3) $$\Delta Perception_{ij}=\beta_{0}+\beta_{1}Knowledge_{i}+\beta_{2}Individual_{i}+\beta_{3}Household_{i}+\beta_{4}Other_{ij}+\varepsilon_{ij}$$

By contrast with Equation (1), the dependent variable in Equation (3), ΔPerception, is the change in consumers’ perceptions, purchase intentions, and consumption of GM food after the COVID-19 outbreak. For simplicity, we classify consumers’ changes into three groups: consumers who become more negative toward GM food (ΔPerception=1), whose attitudes remain unchanged (ΔPerception=0), and whose attitudes become more favorable toward GM food (ΔPerception=−1) after the COVID-19 outbreak. Finally, since the dependent variable measures a change, not a level, *COVID-19* is excluded from Equation (3).

Equation (3) also adds a new explanatory variable, *Knowledge*. We use this variable to measure how well a consumer knows GM technology. To gauge consumers’ knowledge, we include five questions in the questionnaire obtained from the website of the Ministry of Agriculture (http://www.moa.gov.cn/ztzl/zjyqwgz/kpxc/). We record respondents’ answers to each question.^6^^6^We communicate the correct answers after they answer these five questions. The variable, *Knowledge*, reflects the total number of questions that respondents answer correctly.

However, the estimation results of Equation (3) may be biased due to the endogeneity of *Knowledge* caused by omitted variables. To address this issue, we estimate the following model:

$$Knowledge_{ijt}=\gamma_{0}+\gamma_{1}Information_{i}+\gamma_{2}Education_{i}+\gamma_{3}Age_{i}+\gamma_{4}Individual_{i}+\gamma_{5}Household_{i}+\gamma_{6}Other_{ij}+\varsigma_{ij}$$

(4) $$\Delta Perception_{ij}=\theta_{0}+\theta_{1}Knowledge_{i}+\theta_{2}Individual_{i}+\theta_{3}Household_{i}+\theta_{4}Other_{ij}+\tau_{ij}$$

In Equation (4), we use instrumental variables to address the potential endogeneity of consumers’ knowledge. We instrument it by consumers’ information source, education, and age.

The instrumental variable *Information* is a vector variable. Our questionnaire records the sources from which respondents obtain information about GM technology. We classify the information sources into five categories: traditional media, new media, relatives and friends, school, and others. Traditional media include TV, radio, newspapers, and magazines. The official websites of government agencies are also classified as traditional media, as information on GM food on these websites is very similar to that on TV and in newspapers. New media include personal websites and social media, such as Tencent, WeChat, QQ, and Weibo. Hence, we add four information source dummies to the proposed model: new media, relatives and friends, school, and other information sources.

Since the information source is highly correlated with consumers’ education and age, we include these two variables in the instrumental variable equation. In China, GM technology was first included in textbooks for sophomore students in high schools in 2003. Hence, the high school and above education level highly correlates with the school information source. For a similar reason, age is also correlated with school information and internet information sources. Our data also show that age and education are highly correlated with these five information sources.^7^^7^Except for age, the estimated coefficients on the high school and above education dummy and age are all significant with p-values lower than 1% in all the information source equations.

## 3. Results

Table 2 reports the estimation results of Equation (1). The estimation results confirm that consumers’ perceptions and consumption of GM food turn negative after the COVID-19 outbreak. Most estimated coefficients have the expected signs and align with previous studies. For example, the estimated results show that consumers with more knowledge of GM technology are more likely to approve GM food (row 2, Table 2). These findings are consistent with previous studies, such as Cui and Shoemaker, Hursti and Magnusson, and Zhang and Liu.^9,18,19^ In addition, male respondents are more likely to positively perceive GM food, as shown by studies such as Florkowski et al. and Costa-Font et al.^20,21^

Notably, the estimation results indicate that the COVID-19 outbreak significantly impacts consumers’ perceptions and consumption of GM food. The estimated coefficients on *COVID-19* are significant in all three equations (row 1, Table 2). As shown in the first and second columns of Table 2, the coefficients in both the consumers’ perception and purchase intention equations are positive, indicating a worsening in consumers’ perceptions and purchase intentions toward GM food (i.e., opposing and/or strongly opposing GM food). By contrast, the negative coefficient in the actual purchase equation indicates that consumers have purchased less GM food after the COVID-19 outbreak (column 3 of Table 2). This finding is consistent with previous studies (e.g., Ghufran et al.) which showed that the COVID-19 pandemic strongly pushed Chinese consumers’ intentions toward organic food, which is considered safer than traditional food.^22^

Table 3 reports the estimated marginal effect of COVID-19. After the COVID-19 outbreak, the shares of consumers who strongly approve, approve, and are neutral toward GM food have decreased by 0.29%, 0.92%, and 0.43%, respectively (row 1 of Table 3). By contrast, the shares of consumers who oppose and strongly oppose GM food have increased by 0.58% and 1.06%, respectively (column 1 of Table 3). Similarly, the total share of consumers with a positive purchase intention toward GM food has decreased by 0.57% (0.12%+0.45% = 0.57%), while the total percentage of consumers with no intention to purchase GM food has increased by 0.82% (0.32% + 0.50% = 0.82%). Finally, as shown in the last column of Table 3, the number of consumers purchasing GM food has decreased by 3.36% in 2020.

Table 4 reports the estimation results of Equation (2). In line with Table 3, the results in Table 4 confirm the negative shift in consumers’ perceptions and consumption of GM food. As shown in the upper panel of Table 4, the estimated coefficients on *COVID-19* are all statistically significant (row 1, Table 4), indicating that consumers’ perceptions and purchase intentions toward GM food have become more negative after the COVID-19 outbreak.^8^^8^Further research shows that the marginal effects of the COVID-19 outbreak for consumers whose perceptions and purchase intentions of GM food are positive (i.e., strongly approve, approve, and neural) decrease, while the marginal effects for consumers with negative perceptions and purchase intentions of GM food (i.e., oppose and strongly oppose) increase. In addition, the negative coefficient in the actual purchase equation indicates that consumers’ purchase of GM food has decreased in 2020.

**Table 4.** Impact of COVID-19 on consumers’ perceptions and consumption of genetically modified food – individual fixed-effect model.

		Dependent variable		
		perception	purchase intention	Actual purchase
COVID-19 (Yes = 1)		0.4861***	0.1397**	−0.7718***
		(6.32)	(1.97)	(−5.61)
COVID-19*Soybean oil		−0.2307**	−0.0344	0.1879
		(−2.53)	(−0.41)	(1.04)
COVID-19*Tofu		−0.2409***	0.0859	0.5300***
		(−2.74)	(1.03)	(2.91)
Soybean oil dummy		0.3592***	−0.0237	0.9098***
		(3.17)	(−0.21)	(7.56)
Tofu dummy		0.1927*	−0.3169***	0.4307***
		(1.75)	(−2.85)	(3.55)
COVID-19 (Yes = 1)	Papaya	0.8650***	0.2569**	−1.3437***
		(6.26)	(1.96)	(−6.55)
	Soybean oil	0.5497***	0.2387*	−1.3863***
		(3.87)	(1.71)	(−6.68)
	Tofu	0.5596***	0.5517***	−0.6518***
		(3.89)	(3.94)	(−3.21)

Note: Robust z-statistics in parentheses. ***p < .01, **p < .05, *p < .1.

The negative shift in consumers’ perceptions and purchase intentions toward GM food is consistent for all three GM foods. As shown in rows 2 and 3 of Table 4, even though the coefficients on the interaction terms (*COVID-19* and *soybean oil*, and *COVID-19* and *tofu*) are negative in the perception equation, their absolute values are all smaller than the coefficient on *COVID-19* (0.4861 vs. −0.2307 and −0.2409). This result indicates that consumers’ perceptions of soybean oil and tofu have worsened after the COVID-19 outbreak. However, consumers’ purchase intentions show no significant differences between papaya, soybean oil, and tofu (i.e., the estimated coefficients of the interaction terms are insignificant). Finally, consumers’ actual purchases of GM food have decreased, even though the impact on tofu has reduced (last column, Table 4).

To verify the robustness of the estimation results, we rerun the model with three different GM foods. As shown in the lower panel of Table 4, all the estimated coefficients on *COVID-19* are statistically significant. Consumers’ perceptions and purchase intentions of all three GM foods worsen, and their purchases of GM food decrease after the COVID-19 outbreak.

Table 5 reports the estimation results of Equation (3). Knowledge of GM food significantly impacts consumer change. The estimation coefficients on *Knowledge* in both the perception and purchase intention equations are negative and statistically significant (row 1, Table 5). Overall, the estimation results show that consumers with more knowledge of GM technology are less likely to change their perceptions of GM food negatively.

**Table 5.** Impact of COVID-19 on the change in consumers’ perceptions, purchase intentions, and actual purchase.

	Dependent variable: change of		
	perception	purchase intention	actual purchase
Knowledge of GM	−0.1067***	−0.0799***	0.0009
	(−4.33)	(−3.28)	(0.03)
Number of years the consumer has	−0.0198*	−0.0136	0.0069
been aware of GM food	(−1.92)	(−1.33)	(0.53)
Male (1=male)	−0.1103	−0.1253	−0.1712
	(−1.27)	(−1.46)	(−1.52)
Government or state-owned firms	−0.2172	−0.1943	0.0217
(Yes = 1)	(−1.50)	(−1.33)	(0.12)
Private firms (Yes = 1)	−0.3737**	−0.2255	0.2715
	(−1.97)	(−1.20)	(1.14)
Student (Yes = 1)	−0.4308***	−0.3234**	−0.1934
	(−2.71)	(−2.03)	(−0.94)
Agriculture-related work (Yes = 1)	−0.0318	−0.1835	0.1672
	(−0.18)	(−1.03)	(0.74)
Grocery shopping dummy	0.1540*	0.0596	0.1631
	(1.74)	(0.68)	(1.42)
Family size	0.0567*	−0.0048	0.0112
	(1.81)	(−0.15)	(0.27)
Family member with a food allergy	−0.2742**	−0.1821	0.1833
	(−2.25)	(−1.52)	(1.23)
Middle-income (50 ~ 100k) dummy	0.0034	−0.2238**	−0.0569
	(0.03)	(−2.22)	(−0.43)
High-income (>100k) dummy	−0.1356	−0.1684	0.1451
	(−1.29)	(−1.62)	(1.09)
Soybean oil dummy	−0.2249**	−0.0986	0.0235
	(−2.24)	(−0.99)	(0.19)
Tofu dummy	0.0226	−0.0746**	0.4019*
	(0.72)	(−2.16)	(1.89)
City dummies	Yes	Yes	Yes
Constant cut1	−3.8572***	−3.3820***	−4.2545***
	(−15.09)	(−13.33)	(−12.63)
Constant cut2	2.0004***	2.3760***	3.1483***
	(8.18)	(9.57)	(9.69)
Observations	6,090	6,090	6,090

Note: City cluster robust z-statistics in parentheses. ***p < .01, **p < .05, *p < .1. GM: genetically modified.

Table 6 summarizes the marginal effect of consumers’ knowledge on their change. As shown in the first column of Table 6, as consumers’ knowledge increases (i.e., being able to answer one more GM technology question correctly), their perception shift toward approving GM technology or remaining unchanged increases by 0.42% and 0.27%, respectively. In addition, their perception change toward opposing GM technology decreases by 0.68%. Similarly, consumers’ knowledge positively impacts their purchase intentions; Consumers’ shift toward opposing GM technology decreases by 0.46%, while the percentage of consumers who became more approving of GM technology increases by 0.37%.

**Table 6.** Marginal effect of COVID-19 on consumers’ perception change.

	perception	purchase intention
Increase in approval	0.0042***	0.0037***
	(4.20)	(3.24)
Unchanged	0.0027***	0.0009**
	(3.67)	(2.17)
Increase in opposition	−0.0068***	−0.0046***
	(−4.27)	(−3.25)
Observations	6,090	6,090

Note: z-statistics in parentheses. ***p < .01, **p < .05, *p < .1.

Surprisingly, we find no impact of consumers’ knowledge on their actual purchases of GM food. As shown in the first row of Table 5, the estimated coefficient on *Knowledge* in the purchase equation is insignificant. We re-estimate the model with three different GM foods and/or adding a north dummy (which equals one for Beijing, Harbin, and Lanzhou, and zero otherwise) and still obtain insignificant estimates.^9^^9^The north dummy is added to reflect the fact that people in north China consume more soybean oil and less papaya. This finding is in line with previous studies showing that consumers’ purchases are often inconsistent with their reported perceptions and purchase intentions.^23–26^

Table 7 reports the estimation results of Equation (4). First, as shown in the last column of Table 7, the estimated coefficients on the relatives and friends information source and school information source are statistically significant, indicating that these information sources impact consumers’ knowledge of GM technology. We further test the significant impact of all the four estimated coefficients on information source dummies and obtain an F-value of 5.63 with a P-value lower than 1%. These results indicate that information source is a reliable instrumental variable for consumers’ knowledge of GM technology.

**Table 7.** Estimation results of the impact of knowledge on consumer change.

	Dependent variable			
	perception	purchase intention	actual purchase	knowledge
Knowledge	−0.0384***	−0.0395***	−0.0043	
	(−3.17)	(−3.49)	(−0.63)	
New media information source				−0.0018
				(−0.04)
Relatives and friends source				−0.2018***
				(−2.77)
School information source				0.4014***
				(3.55)
Other information sources				−0.0634
				(−0.49)
Age				−0.0361***
				(−18.30)
High school and above dummy				0.2411***
				(4.28)
Male	−0.0097	−0.0146	−0.0107	−0.0766*
	(−0.82)	(−1.31)	(−1.59)	(−1.73)
Number of years the consumer has	−0.0025*	−0.0024*	0.0004	0.0022
been aware of GM technology	(−1.74)	(−1.84)	(0.44)	(0.41)
Government or state-owned firms	−0.0448**	−0.0390**	−0.0007	−0.2885***
	(−2.20)	(−2.05)	(−0.06)	(−3.83)
Private firms	−0.0378	−0.0144	0.0188	0.2199**
	(−1.40)	(−0.57)	(1.23)	(2.22)
Student	−0.0767***	−0.0625***	−0.0150	−0.1672*
	(−3.29)	(−2.87)	(−1.13)	(−1.96)
Agriculture-related work	−0.0170	−0.0252	0.0082	−0.1320
	(−0.70)	(−1.11)	(0.59)	(−1.46)
Grocery shopping	0.0137	0.0020	0.0086	−0.0787*
	(1.12)	(0.18)	(1.24)	(−1.74)
Family size	0.0099**	0.0009	0.0006	0.0091
	(2.27)	(0.22)	(0.26)	(0.57)
Family member with a food allergy	−0.0348**	−0.0227	0.0112	−0.0656
	(−2.13)	(−1.49)	(1.21)	(−1.08)
Middle-income group (50k ~ 100k)	−0.0033	−0.0239*	−0.0029	0.0150
	(−0.24)	(−1.84)	(−0.37)	(0.29)
High-income group (>100k)	−0.0158	−0.0100	0.0102	0.0980*
	(−1.07)	(−0.73)	(1.23)	(1.81)
Soybean oil dummy	−0.0325**	−0.0059	0.0015	0.0000
	(−2.38)	(−0.46)	(0.19)	(0.00)
Tofu dummy	−0.0325**	0.0123	−0.0251***	0.0000
	(−2.38)	(0.96)	(−3.24)	(0.00)
Nine city dummies	Yes	Yes		Yes
Constant	0.1605***	0.1212***	0.0366	2.9055***
	(4.06)	(3.28)	(1.63)	(18.75)
*N*	6090	6090	6090	6090

Note: t statistics in parentheses. *p < .10, **p < .05, ***p < .01. GM: genetically modified.

Table 7 confirms the impact of consumers’ knowledge on their perceptions and purchase intentions of GM food. As shown in the first row of Table 7, the estimated coefficients on *Knowledge* are negative and statistically significant, indicating that the more knowledge consumers have, the less likely they are to change their perceptions and purchase intentions of GM food, assuming a more negative attitude toward GM technology. In line with Table 5, the impact of knowledge on consumers’ actual purchases is insignificant, confirming that consumers’ perception change may differ from their consumption behavior (column 3, Table 7).

## 4. Policy Implications

The results of this study have meaningful policy implications. First, China faces a significant challenge in terms of reversing consumers’ perceptions of GM food after the COVID-19 outbreak. Previous studies have shown that Chinese consumers’ perceptions of GM food turned positive to negative a decade ago.^9,10^ Our data indicate that 21% of consumers have a positive perception of GM food, while 40% have a negative perception. In addition, this study further shows that consumers’ perceptions and consumption have deteriorated after the COVID-19 outbreak. Due to the country’s limited resources and increasing demand, Chinese authorities must accelerate the commercialization of GM crops.^27^ However, due to a rise in opposition to genetic modification technology in the past decade, the commercialization of GM crops has been postponed indefinitely,^28^ even though a million tons of GM soybeans and maize are imported each year.^29^ To accelerate the commercialization of GM crops, Chinese authorities must first reverse consumers’ perceptions, which may now require more effort than before the COVID-19 outbreak.

Second, government authorities should put in greater effort educating the public about genetic modification technology. Previous studies have shown that consumers’ knowledge of GM technology highly correlates with their perceptions.^14,18^ This study finds that the more knowledge consumers have, the less likely they are to shaft toward a negative perception of GM food (i.e., opposing GM food). Hence, Chinese authorities should focus on educating the public, encouraging scientists and experts to work with traditional and new media.

## 5. Conclusions

Using data from 2030 households in urban China in 2020, this study shows that the COVID-19 outbreak has significantly affected consumers’ perceptions and consumption of GM food. Specifically, consumers’ perceptions have become more negative, and consumers have purchased less GM food after the COVID-19 outbreak, further deteriorating their approval of GM technology. In addition, this study finds that consumers with more knowledge of GM technology are less likely to change their perceptions negatively and decrease their purchase intentions of GM food. In other words, the more knowledge of GM technology consumers have, the less likely they are affected by the COVID-19 outbreak.

We conducted the field survey in November 2020, almost one year after the COVID-19 outbreak. However, the impact of COVID-19 may keep increasing as the pandemic is not over yet. Its effect may become more significant as more people experience the consequences of COVID-19.^30^ Hence, this study’s estimates should be considered as the short-run impact of COVID-19. To comprehensively assess its long-term impact, further research is needed.

## Data Availability

The data that support the findings of this study are available on request from the corresponding author.
